# Synthesis of benzylidenemalononitrile by Knoevenagel condensation through monodisperse carbon nanotube-based NiCu nanohybrids

**DOI:** 10.1038/s41598-020-69764-8

**Published:** 2020-07-29

**Authors:** Nursefa Zengin, Hakan Burhan, Aysun Şavk, Haydar Göksu, Fatih Şen

**Affiliations:** 1 Kaynasli Vocational College, Duzce University, Düzce, 81900 Turkey; 2 Sen Research Group, Biochemistry Department, Faculty of Arts and Science, Dumlupınar University, Evliya Çelebi Campus, 43100 Kütahya, Turkey

**Keywords:** Biochemistry, Catalysis

## Abstract

Monodisperse nickel/copper nanohybrids (NiCu@MWCNT) based on multi-walled carbon nanotubes (MWCNT) were prepared for the Knoevenagel condensation of aryl and aliphatic aldehydes. The synthesis of these nanohybrids was carried out by the ultrasonic hydroxide assisted reduction method. NiCu@MWCNT nanohybrids were characterized by analytical techniques such as X-ray diffraction (XRD), X-ray photoelectron spectroscopy (XPS), transmission electron microscopy (TEM), high-resolution transmission electron microscopy (HR-TEM), and Raman spectroscopy. According to characterization results, NiCu@MWCNT showed that these nanohybrids form highly uniform, crystalline, monodisperse, colloidally stable NiCu@MWCNT nanohybrids were successfully synthesized. Thereafter, a model reaction was carried out to obtain benzylidenemalononitrile derivatives using NiCu@MWCNT as a catalyst, and showed high catalytic performance under mild conditions over 10–180 min.

## Introduction

Benzylidenemalononitrile (BMN) derivatives are frequently used as both a target molecule and an intermediate molecule in organic chemistry. BMN derivatives have attracted many scientists' attention because of some unique properties such as anticancer^1–3^, antifungal^4,5^, antibacterial^6–9^, anti-corrosive^10^. BMN derivatives are used to increase cell resistance in the case of oxidative stress^11^, in prostaglandin biosynthesis^12^, in the design of photoconductive cells^13^ and the inhibition/activation of certain enzymes^14,15^.

**Figure 1:**
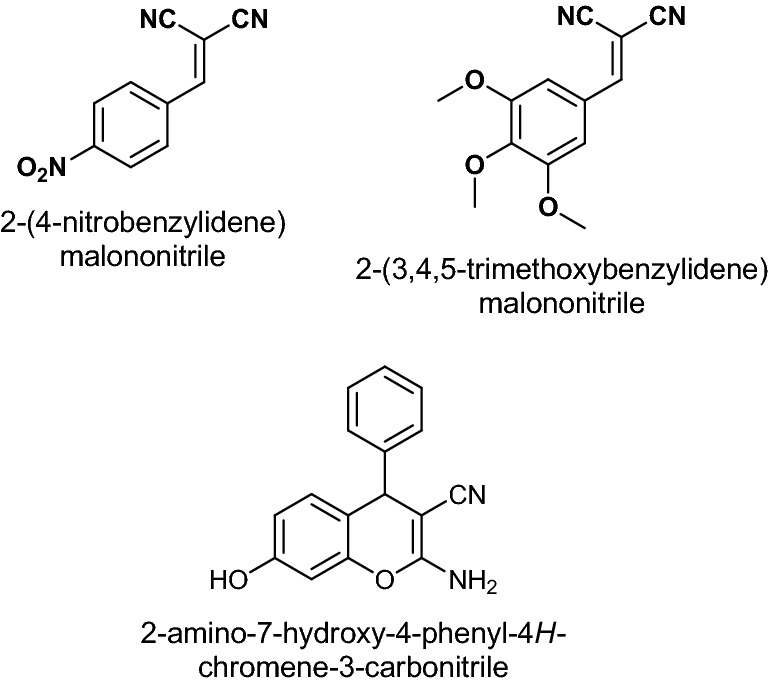
Benzylidenemalononitrile derivatives.

Typically, BMN derivatives are synthesized by the Knoevenagel condensation of aldehydes with active methylene compounds (Fig. 1)^16–22^. In these processes, it is seen that it has an effect on homogeneous catalysts. Heterogeneous catalysts have various advantages such as minimum availability, recovery, reusability, structural deterioration resistance and leaching of metal values^23–28^. Bimetallic heterogeneous catalysts, in particular, have greatly facilitated the Knoevenagel condensation reaction^29–34^. In literature, many catalysts such as SBA-​15 (highly stable mesoporous silica)^35^, Sr_3_Al_2_O_6_ nanoparticles^36^, bifunctional MIL-101(Cr)^37^, Fe_3_O_4_@SiO_2_ nanoparticles^38^ and Mn-MOF@Pi^39^ have been used in condensation reactions.

Herein we report an eco-friendly and practical method for the synthesis of highly efficient, cost-effective, and monodisperse bimetallic NiCu@MWCNT nanohybrids for the Knoevenagel condensation reaction under mild conditions. BMN derivatives have been successfully obtained in a very short time (10–180 min) using the developed method in the water-containing solvent system.

## Experimental

### Chemicals

NiCl_2_, CuCl_2_, NaBH_4_, aminoborane aryl azides, acetonitrile (ACN), lithium perchlorate (LiClO_4_), 3,4-ethylene dioxythiophene (EDOT), multiwall carbon nanotubes, NN –Dimethylformamide (DMF), sulfuric acid, hydrochloric acid, and nitric acid were purchased from Sigma-Aldrich. Tetrahydrofuran (THF; 99.5%), HClO_4_ (60%), 2-propanol, and Methanol (≥ 99.5%) were obtained from Merck. All test materials and other components were cleaned with distilled water. The pure water used was supplied by the Millipore water distillation system.

### The preparation of monodisperse NiCu@MWCNT nanohybrids

NiCu@MWCNT nanohybrids were synthesized using the new ultrasonic hydroxide assisted descent method. 5.25 mg/mL MWCNT, 0.25 mmol CuCl_2,_ and NiCl_2_ precursors were combined in 20 mL water, followed by synthesis under an ultrasonic tip sonicator for 1 h. Finally, 20 mg of sodium borohydride solution was added. The generation of the monodisperse NiCu@MWCNT nanohybrids was observed with the formation of black color. The obtained NiCu@MWCNT nanohybrids were dried in a vacuum oven at 25 °C. Monodisperse NiCu@MWCNT nanohybrids were synthesized and characterized by Raman spectroscopy, XPS, TEM, HRTEM, and XRD techniques. After the characterization of nanohybrids, the catalytic performance of BMN derivatives was investigated.

### Preparation and application of the Knoevenagel condensation studies of aryl and aliphatic aldehydes

4 ml of water/methanol (1:1) solution containing 4 mg NiCu@MWCNT nanohybrids was placed in an ultrasonic bath for 30 s, and at room temperature 1.0 mmol of malononitrile and 1.0 mmol of aldehydes were transferred into the resulting solution (during stirring), and then the slurry was closed. During the mixing, the products formed in the reaction occurred were examined by thin-layer chromatography (TLC). The completion of the entire reaction time ended within 10–180 min, and centrifugation (7,500 rpm) was performed to remove the nanohybrid. The obtained nanohybrid was washed several times using water and methanol, dried in vacuum at room temperature. The purification of the solid sample was done using a chromatography system containing a ratio of 1:9 EtOAc/hexane. ^1^H and ^13^C NMR spectra were taken with deuterated solvents and products were determined using spectra.

## Results and discussion

**Figure 2:**
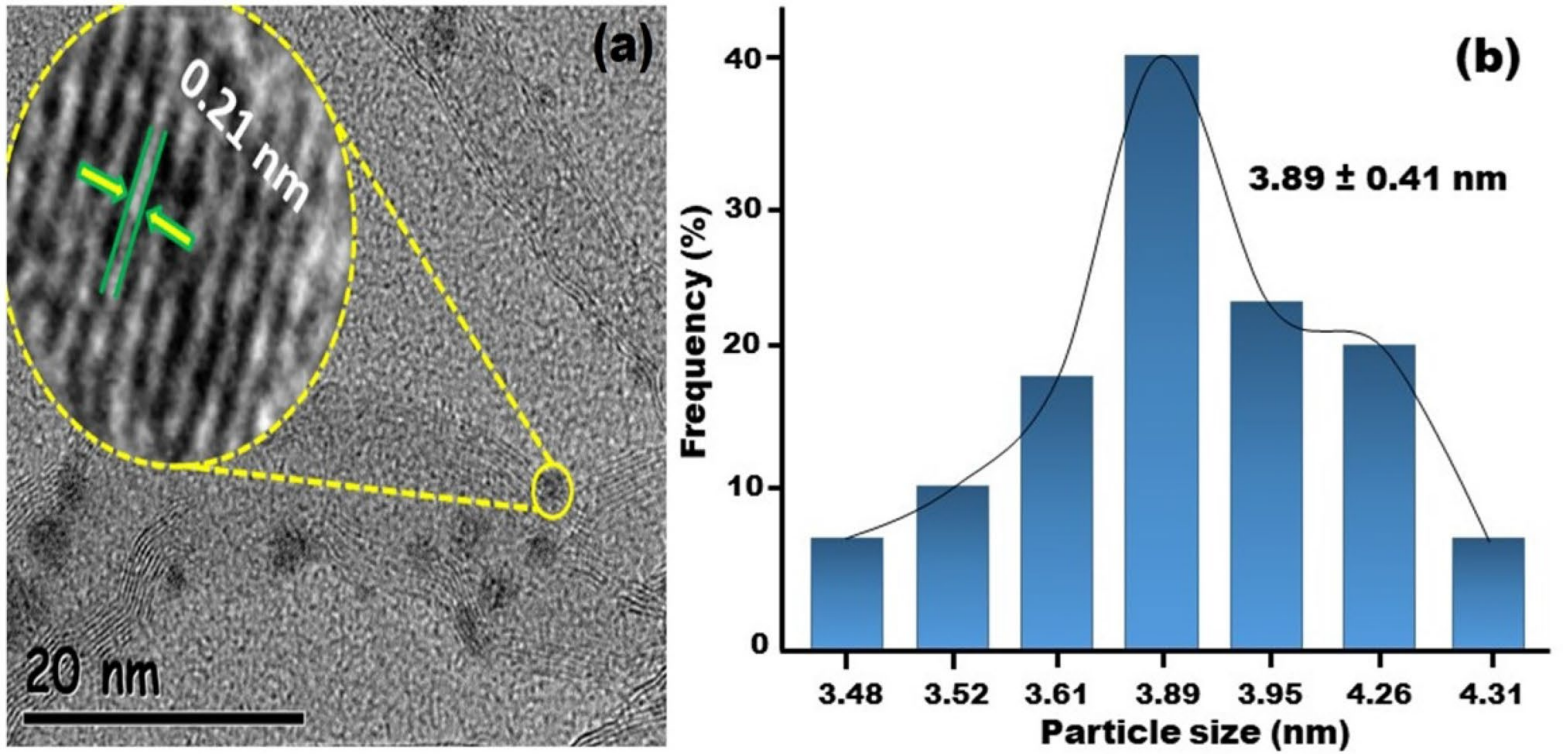
(**a**) Distribution of NiCu@MWCNT monodisperse TEM and HR-TEM analysis (**b**) histogram of mean particle size of monodisperse NiCu@MWCNT.

The morphology and metal nanoparticles distribution on the NiCu@MWCNT nanohybrids were investigated using HR-TEM and TEM analysis. Figure 2a reveals the uniform dispersion of nickel and copper on multiwalled carbon nanotube and homogeneous catalyst structure. The lattice fringe of the NiCu@MWCNT nanohybrids was investigated with HRTEM analysis. A lattice fringe of 0.21 nm corresponding to NiCu (111) was detected on the surface of NiCu@MWCNT nanohybrids. This value is very close to the nickel nominal value of 0.20. This smaller value of the atomic lattice fringe of NiCu nanohybrids compared to the nominal value can be explained by the alloy formation on the catalyst surface. Further, the average particle size of prepared nanohybrid was calculated by accounting for almost 100 particles and, a histogram was given in Fig. 2b. The average particle size of monodisperse NiCu@MWCNT nanohybrids was found to be 3.89 ± 0.41 nm.

The Raman analysis in Fig. S1a shows the ratio of the intensity of D-G bands (I_D_/I_G_) for NiCu@MWCNT nanohybrids and MWCNT performed by Raman spectroscopy. MWCNTs are cylindrical nanoparticles combined with multiple graphene layers with their shapes, sizes, and unusual physical properties. In addition, MWCNTs act as a scaffold for the insertion of many metal nanoparticles due to their large surface area^40–42^. The ratios of I_D_/I_G_ for NiCu@MWCNT nanohybrids and MWCNT were found to be 1.38 and 1.22, respectively. These values show the functionalization and/or defects of multiwalled carbon nanotubes with the help of NiCu nanohybrids. In addition, peak positions of D and G band in the Raman spectrum for NiCu@MWCNT nanohybrid were found as 1,350 and 1,578 cm^-1^. The XRD pattern of the as-synthesized NiCu@MWCNT catalyst was shown in Fig. S1b. It was observed that the XRD pattern consists of well-separated peaks which indicate a face-centered cubic (fcc) crystal lattice structure. The diffraction peaks detected at 2θ degrees of 42.5°, 61.3°, and 73.5° correspond to planes of (111), (220), and (311), respectively. Furthermore, the peak at 2θ degree of 25.6° (002) specified for MWCNT.

The XPS analysis of the monodisperse NiCu@MWCNT nanohybrids is given in Fig. S2. As shown in Fig. S2a, Cu 2*p*_1/2,_ and 2*p*_3/2_ signals can be seen easily and corresponded to the doublets at around 951.6–931.8 and 954.2–934.3 eV, respectively. These values can be ascribed to Cu(II) and Cu(0). Mostly, the formation of Cu(0) and the smaller amount of the formation of Cu(II) were observed. This indicates the formation of NiCu nanohybrids. The smaller amount of Cu(II) most probably comes from some of the unreduced and/or oxidized Cu species. At the same time, the XPS spectra of the Cu 2*p* region show the characteristic shake-up satellites related to CuO and most likely CuO·H_2_O arising from the reaction between Cu(II) and the etchant O. The XPS analysis was also examined for Ni 2*p*_3/2_ region which is given in Fig. S2(b) and its core level were found to be 853.2 eV. The reduction of Ni (II) to Ni (0) was occurred during the synthesis process according to the XPS results (Fig. S2b), namely most of the nickel atoms on the surfaces of NiCu@MWCNT nanohybrids have a metallic structure. The other peak at 858. 5 eV as shown in Fig. S2(b) shows the formation of a small amount of Ni (II) which is mostly coming from an oxidation or chemical desorption in the catalyst composition during the preparation of the catalyst. After full characterization of monodisperse NiCu@MWCNT nanohybrids, they were performed for Knoevenagel condensation reaction as a model reaction to see the effectiveness of the nanohybrids. Generally, we did not test with solvents outside methanol and water, considering previous work by our group on Knoevenagel condensation. In addition to using methanol and water separately (Table 1, entries 1 and 2), we also used it as a mixture (Table 1, entries 3–6). As can be seen, the water–methanol mixture is more effective in the reaction. The prepared sample containing 4 mg NiCu@MWCNT nanohybrids, 1.0 mmol malononitrile, 1.0 mmol 4-iodobenzaldehyde, and 4 ml solution of water/methanol (v/v = 1/1) gave a very high activity for the Knoevenagel condensation reaction. The results are given in Table 1. No Knoevenagel condensation reaction took place without catalyst.

**Table 1:** Some solvents and catalysts used for the model reaction of 4-iodobenzaldehyde with malononitrile^a^.


Entry	Solvent	Time (min)	Catalyst (mg)	Yield^b^ (%)
1	MeOH	120	4	60 ± 2
2	H_2_O	120	4	28 ± 3
3	H_2_O/MeOH (2:1)	30	4	85 ± 2
4	H_2_O/MeOH (3:1)	30	4	72 ± 1
5	H_2_O/MeOH (1:1)	25	4	96 ± 3
6	H_2_O/MeOH (1:1)	240	-	Trace

^a^Reaction conditions: 4-iodoobenzaldehyde (1.0 mmol), malononitrile (1.0 mmol), NiCu@MWCNT nanohybrids (%9.2 wt metal content) and room temperature.

^b^Isolated yield.

The catalytic efficiency of the NiCu@MWCNT nanohybrids was relatively high compared to that reported in previously published works dealing with the Knoevenagel condensation of benzaldehyde with malononitrile (Table S1).

We tried to summarize all of the aryl and aliphatic aldehydes in Table 2 in conjunction with malononitrile which was successfully used to convert them to BMN compounds by NiCu@MWCNT nanohybrids. Obtaining BMN species at 25 °C in an aqueous solution was carried out quantitatively in 10–180 min. The electron-withdrawing groups linked to the aromatic aldehyde molecules increase the efficiency of the condensation reaction. Table 2, entry 1: 3,4,5-trimethoxybenzaldehyde (1) was converted to 2-(3,4,5-trimethoxybenzylidene) malononitrile (2) with the quantitative yield (96 ± 2%) within 10 min. Table 2, entry 2: 4-hydroxybenzaldehyde (3) was converted to 2-(4-hydroxybenzylidene) malononitrile (4) with quantitative yields (95 ± 1%) within 12 min. Table 2, entry 3, 4: 2-(4-methylbenzylidene) malononitrile (6) and 2-(2-methylbenzylidene) malononitrile (8) were obtained with quantitative yields (95 ± 3%) within 15 min. Although the methoxy, hydroxy, and methyl groups are electron donor groups, the reaction for Table 2 entry 1 was completed earlier (Table 2, entry 2). Methyl groups inductively donate electrons to the aromatic ring. The *ortho* and *para-*tolualdahyde were converted to the corresponding BMN derivatives with high yields (Table 2, entries 3, 4). 2-(4-nitrobenzylidene) malononitrile (10) and 2-(4-(trifluoromethyl) benzylidene) malononitrile (12) were obtained with a yield of 96 ± 1%. Nitro (-NO_2_) and trifluoromethyl (-CF_3_) groups are electron-withdrawing groups so that the reactions are completed in a shorter time (Table 2, entries 5, 6).

**Table 2:**
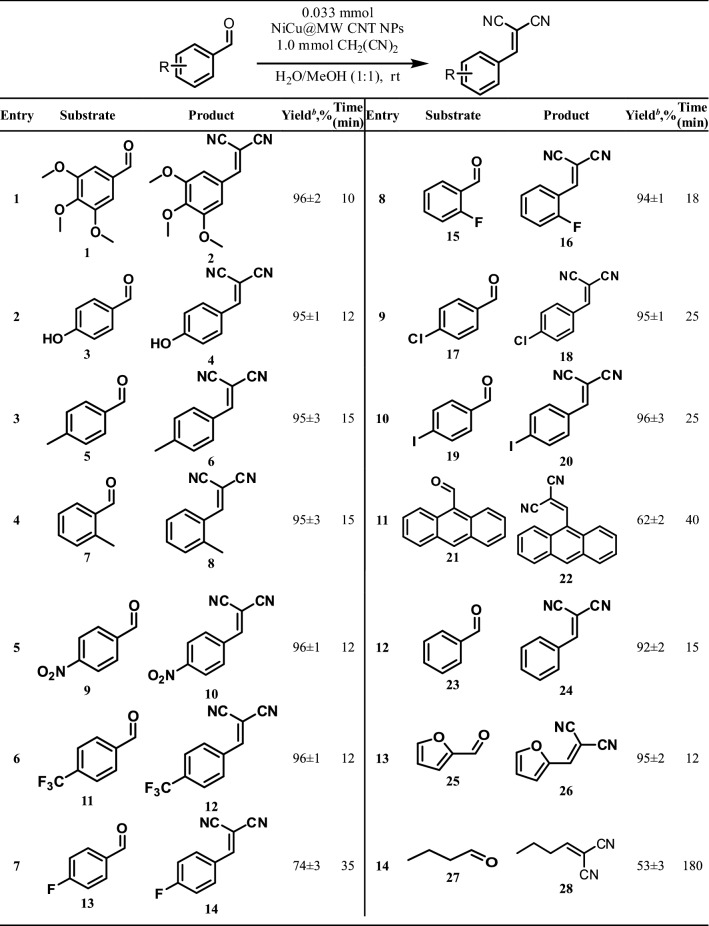
NiCu@MWCNT nanohybrids catalyzed Knoevenagel condensation reaction for different aryl aldehydes and malononitriles^a^.

^a^Reaction conditions: Substrate (1.0 mmol), malononitrile (1.0 mmol) and NiCu@MWCNT (4 mg, %9.2 wt amount of metal) was used with 4 mL of H_2_O/CH_3_OH (v/v = 1/1) at 25 °C.

^b^Isolated yield.

The *p*-chloro- and iodo benzaldehydes (17 and 19) were respectively converted to 2-(4-chlorobenzylidene) malononitrile (18) and 2-(4-iodobenzylidene) malononitrile (20) with high yields (Table 2, entries 9, 10). But, some changes in reaction time and yield are observed due to the fluorine atom attached to the para position. 2-(4-fluorobenzylidene) malononitrile (14) was obtained with a 74 ± 3% yield within 35 min (Table 2, entry 7). There is an overlap agreement between fluorine-carbon due to its p-orbital dimensions. This is a known fact. Therefore, compared to other halogen atoms, it is susceptible to electron mesomerically. This reduces the reactivity of the carbonyl group^43^.

The 2-fluorobenzaldehyde (15) was converted to 2-(2-fluorobenzylidene)malononitrile (16) with a 94 ± 1% yield because of the electronegative effect of the flour atom (Table 2, entry 8).

2-(anthracen-9-ylmethylene) malononitrile (22) was successfully synthesized, but with yields lower than the other aryl aldehydes. Moreover, the completion of the process took longer than expected, perhaps due to the steric effect (Table 2, entry 11). The benzaldehyde (23) was converted to 2-benzylidenemalononitrile (24) within 15 min with 92 ± 2% yield (Table 2, entry 12).

2-(2-Furanylmethylene) malononitrile (26) was obtained in quantitative yields and in a short time using furan-2-carbaldehyde (25), which is heteroaromatic aldehyde (Table 2, entry 13). On the other hand, this reaction was carried out with aliphatic aldehydes to compare with aromatic aldehydes. But the major disadvantages in using the aliphatic aldehydes as the starting compound were reaction time and efficiency. 2-Butylidenemalononitrile (28) was obtained with 53 ± 3% yield within 180 min (Table 2, entry 14).

The reusability performance of monodisperse NiCu@MWCNT nanohybrids has also been studied, and it can be seen that monodisperse NiCu@MWCNT nanohybrids retain their initial activity even during the fifth reuse (Table 3).

**Table 3:**
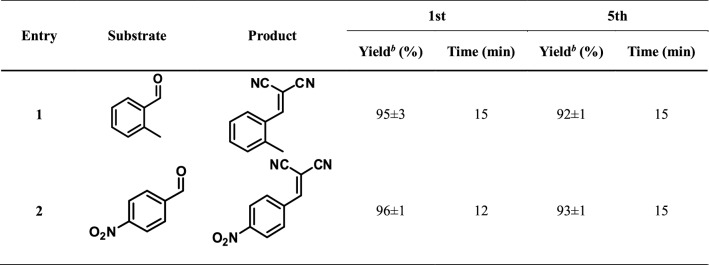
Reusability performance of NiCu@MWCNT nanohybrids^a^.

^a^Reaction conditions: substrate (1.0 mmol), malononitrile (1.0 mmol) and NiCu@MWCNT nanohybrids (4 mg, %9.2 wt amount of metal), 4 mL of H_2_O/CH_3_OH (v/v = 1/1) at 25 °C.

^b^Isolated yield.

## Conclusions

In conclusion, one-pot, practically, the eco-friendly and recoverable synthetic process has been described for the synthesis of BMN derivatives via Knoevenagel condensation with highly monodisperse NiCu@MWCNT nanohybrids. In the synthesis of NiCu@MWCNT nanohybrids, the new ultrasonic hydroxide assisted reduction method was used, where small monodisperse nano-hybrids were obtained without agglomeration problems. NiCu@MWCNT nanohybrids were identified by Raman spectroscopy, XPS, TEM, HR-TEM, and XRD. All characterization techniques showed the alloy formation of the NiCu@MWCNT nanohybrids. Monodisperse NiCu@MWCNT nanohybrids displayed an outstanding catalytic performance for the model reaction. The catalytic activity of this system was compared with those of the previous studies and found to be one of the highest performance and the best catalytic efficiency because of the formation of much smaller and the monodisperse nanohybrids on MWCNT with the help of OH- ligands and ultrasonication process. This case can also be explained by the higher stability, larger active chemical surface area, more metallic contents of Ni and Cu, and smaller particle size for the prepared catalyst compared to the others for the model reaction ^43–49^. The obtained NiCu@MWCNT nanohybrids have unique properties such as efficient, safe, economically, and environmentally friendly at room temperature. Due to these distinguishing features, the obtained NiCu@MWCNT nanohybrids will be highly preferred material in future works for the Knoevenagel reaction and will give a new perspective. The effectiveness of the applied method is that it provides a single pot synthesis to facilitate functionalized carbon-based work with MWCNT. This process of heterogeneous catalysts and Knoevenagel condensation is of great importance.

## Supplementary information

Supplementary file1 (DOCX 2619 kb)
