# Supplementary material for: Synthesis of benzylidenemalononitrile by Knoevenagel condensation through monodisperse carbon nanotube-based NiCu nanohybrids
Source: Sci Rep. 2020 Jul 29;10:12758. doi: 10.1038/s41598-020-69764-8 (PMC7391679; doi:10.1038/s41598-020-69764-8)
Supplement: Supplementary file 1 — Supplementary file1 (DOCX 2619 kb) [file 41598_2020_69764_MOESM1_ESM.docx]

Supplementary Material

Synthesis of benzylidenemalononitrile by Knoevenagel condensation through monodisperse carbon nanotube-based NiCu nanohybrids

Nursefa Zengin^a^, Hakan Burhan^b^, Aysun Şavk^b^, Haydar Göksu^a*^ and Fatih Şen^b*^

^a^ Kaynasli Vocational College, Duzce University, Düzce 81900, Turkey

^b^ Sen Research Group, Biochemistry Department, Faculty of Arts and Science, Dumlupınar University, Evliya Çelebi Campus, 43100 Kütahya, Turkey

**Methods**

**The characterization of NiCu@MWCNT nanohybrids**

The characterization of monodisperse NiCu@MWCNT nanohybrids was performed with the help of some of the advanced analytical techniques such as TEM, XRD, XPS, Raman Spectroscopy, etc. TEM (Transmission Electron Microscopy) analysis of NiCu@MWCNT nanohybrids has been obtained by a JEOL 200 kV TEM instrument. Sample preparation was carried out through the suspension of about 0.5 mg catalyst in 3 ml of ethanol in an ultrasonic bath, and a drop of the resulting solution was placed on a copper grid made of 400 mesh and carbon. Almost 100 particles were investigated to obtain the average particle size and distribution. The drying of the catalyst was carried out at 25 ^o^C. XRD (X-ray diffraction) analysis was performed to investigate the crystal structure of the samples of the NiCu@MWCNT nanohybrids. XRD analysis was done using the Panalytical Empyrean Diffractometer apparatus, X-ray device (λ = 1.54056Å, Cu K radiation) at 40 mA - 45 Kv conditions. Oxidation levels of nickel and copper metals in the NiCu@MWCNT nanohybrids and the surface composition of the NiCu@MWCNT nanohybrids were investigated by XPS analysis using X-ray photoelectron spectrometer having X-ray source at 1253.6 eV, 10 mA on K lines of Mg. A Gaussian function was used to fit peak, and the C 1s line at 284.6 eV was taken as a reference for all the lines. The 2p region for Ni was investigated according to Gaussian – Lorentzian, and the relative intensity for the species was evaluated by counting every peak after subtraction and smoothing of the Shirley – shape background.


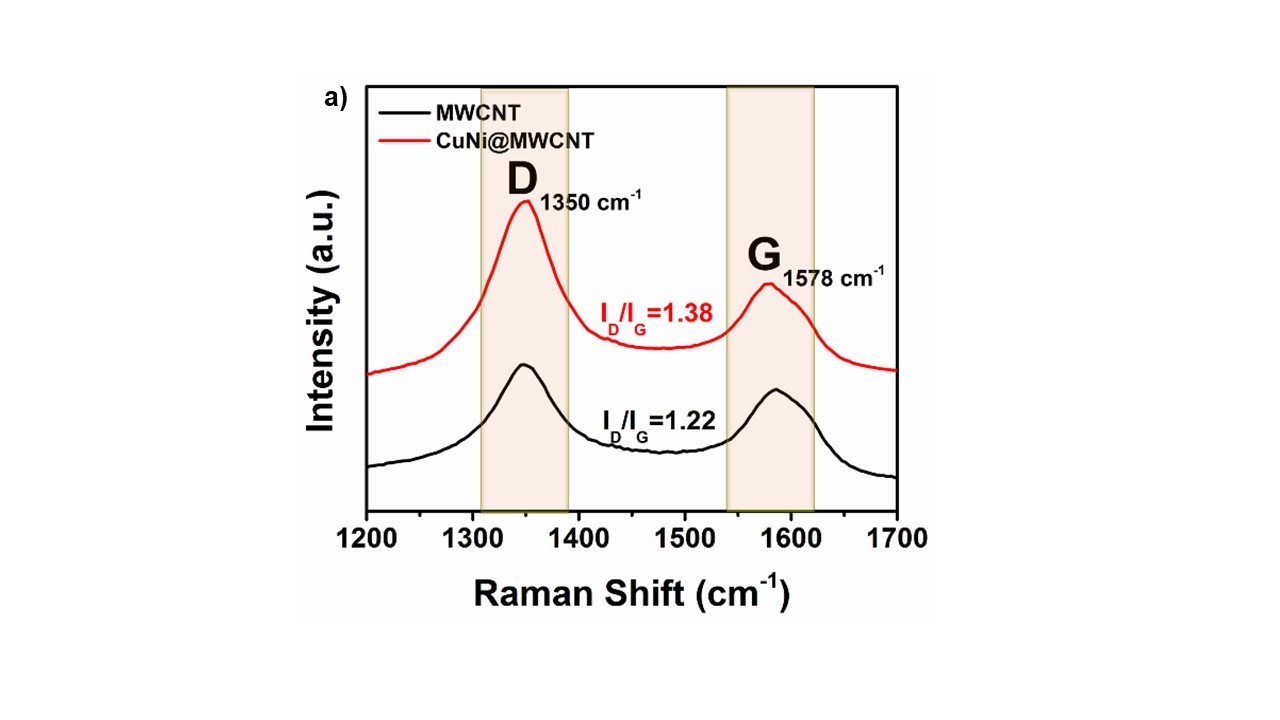


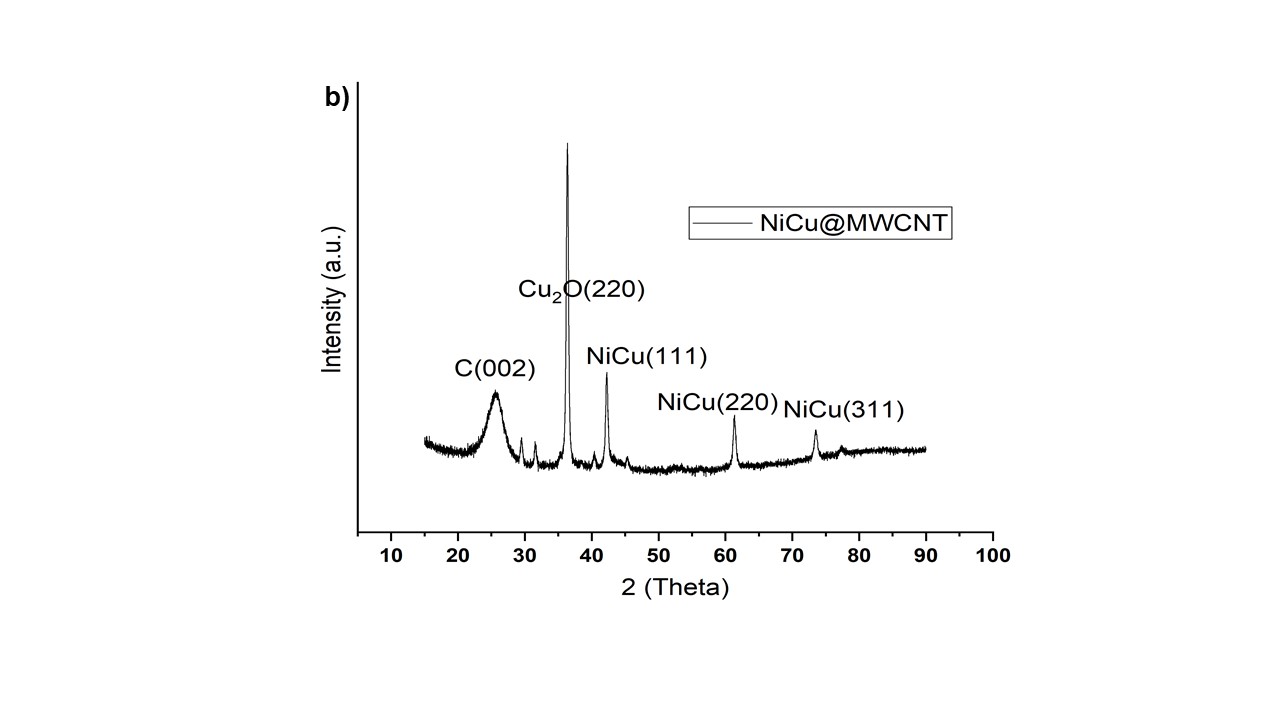


**Figure S1.** (a) Raman spectra of MWCNT and NiCu@MWCNT nanohybrids and (b) XRD pattern of NiCu@MWCNT nanohybrids


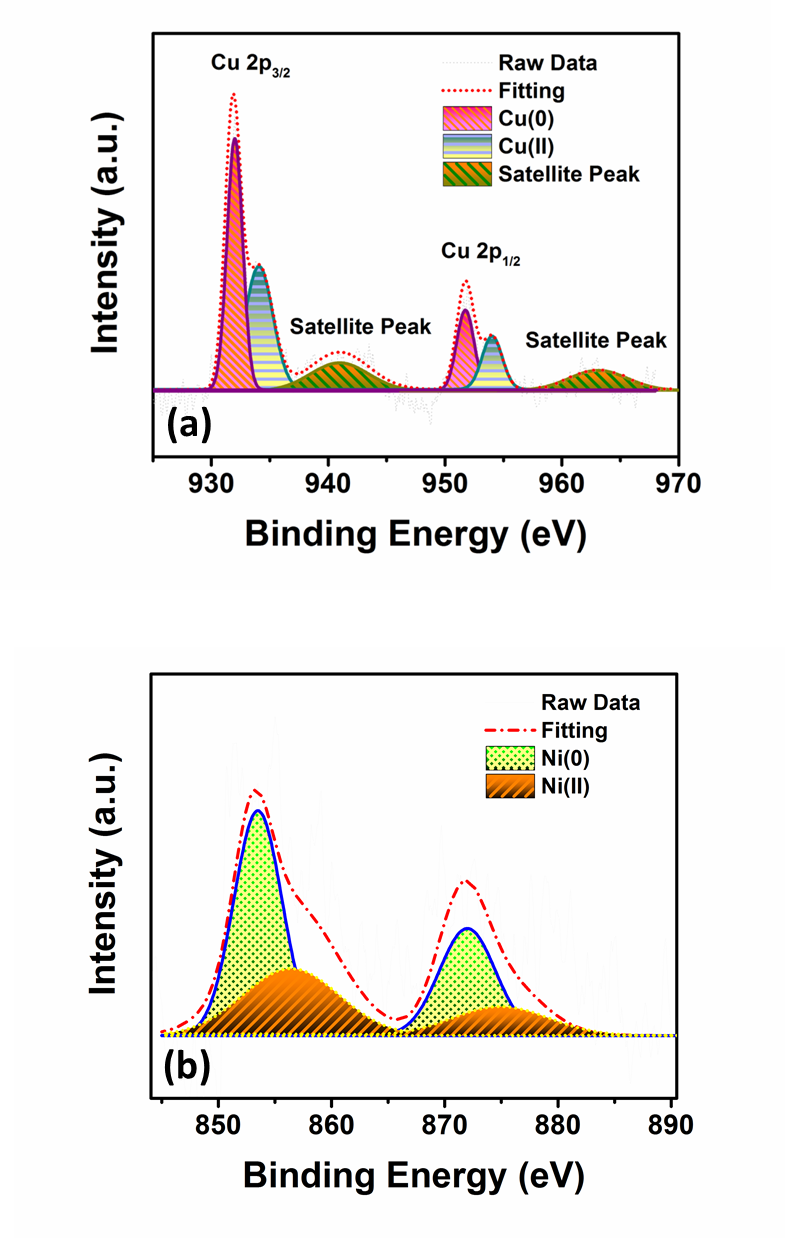


**Figure S2.** XPS spectra (a) Cu 2p region and (b) Ni 2p region of NiCu@MWCNT nanohybrids.

| **EDAX TEAM** | | | | | | | | | | | | | | | | | | | | | | | | | | | | | | | | | | | | | |  |  |  |  |  |
| --- | --- | --- | --- | --- | --- | --- | --- | --- | --- | --- | --- | --- | --- | --- | --- | --- | --- | --- | --- | --- | --- | --- | --- | --- | --- | --- | --- | --- | --- | --- | --- | --- | --- | --- | --- | --- | --- | --- | --- | --- | --- | --- |
|  |  |  |  |  |  |  |  |  |  |  |  |  |  |  |  |  |  |  |  |  |  |  |  |  |  |  |  |  |  |  |  |  |  |  |  |  |  |  |  |  |  |  |
|  | | |  |  |  | | |  | |  | | |  | | | | |  |  |  |  |  |  |  |  |  |  |  |  |  |  |  |  |  |  |  |  |  |  |  |  |  |
|  | | |  |  |  | | |  | |  | | |  | | | | |  |  |  |  |  |  |  |  |  |  |  |  |  |  |  |  |  |  |  |  |  |  |  |  |  |
| 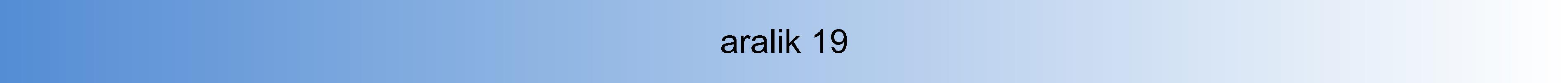 | | | | | | | | | | | | | | | | | | | | | | | | | | | | | | | | | | | | | | | | | | |
|  | | |  |  |  | | |  | |  | | | | | | | | | | | | | | | | | | | | | | | | |  |  |  |  |  |  |  |  |
|  | | |  |  |  | | |  | |  |  |  |  |  |  |  |  |  |  |  |  |  |  |  |  |  |  |  |  |  |  |  |  |  |  |  |  |  |  |  |  |  |
|  | | |  |  |  | | |  | |  |  |  |  |  |  |  |  |  |  |  |  |  |  |  |  |  |  |  |  |  |  |  |  |  |  |  |  |  |  |  |  |  |
|  | | |  |  |  | | |  | | 03/12/2020 2:20:58 PM | | | | | | | | | | | | | | | | | | |  |  |  |  |  |  |  |  |  |  |  |  |  |  |
|  | | |  |  |  | | |  | |  | | |  | | | | |  |  |  |  |  |  |  |  |  |  |  |  |  |  |  |  |  |  |  |  |  |  |  |  |  |
| Sample Name: | | | | | | | | | | | | | |  | |  |  | | | | | | | | | | | | | | | | | | | | | | |  |  |  |
|  | | |  |  |  | | |  | |  | | |  | | | | |  |  |  |  |  |  |  |  |  |  |  |  |  |  |  |  |  |  |  |  |  |  |  |  |  |
| **Area 297** | | | | | | | | | | | | | | | | | | | | | | | | | | | | | | | | | | | | | | |  |  |  |  |
| 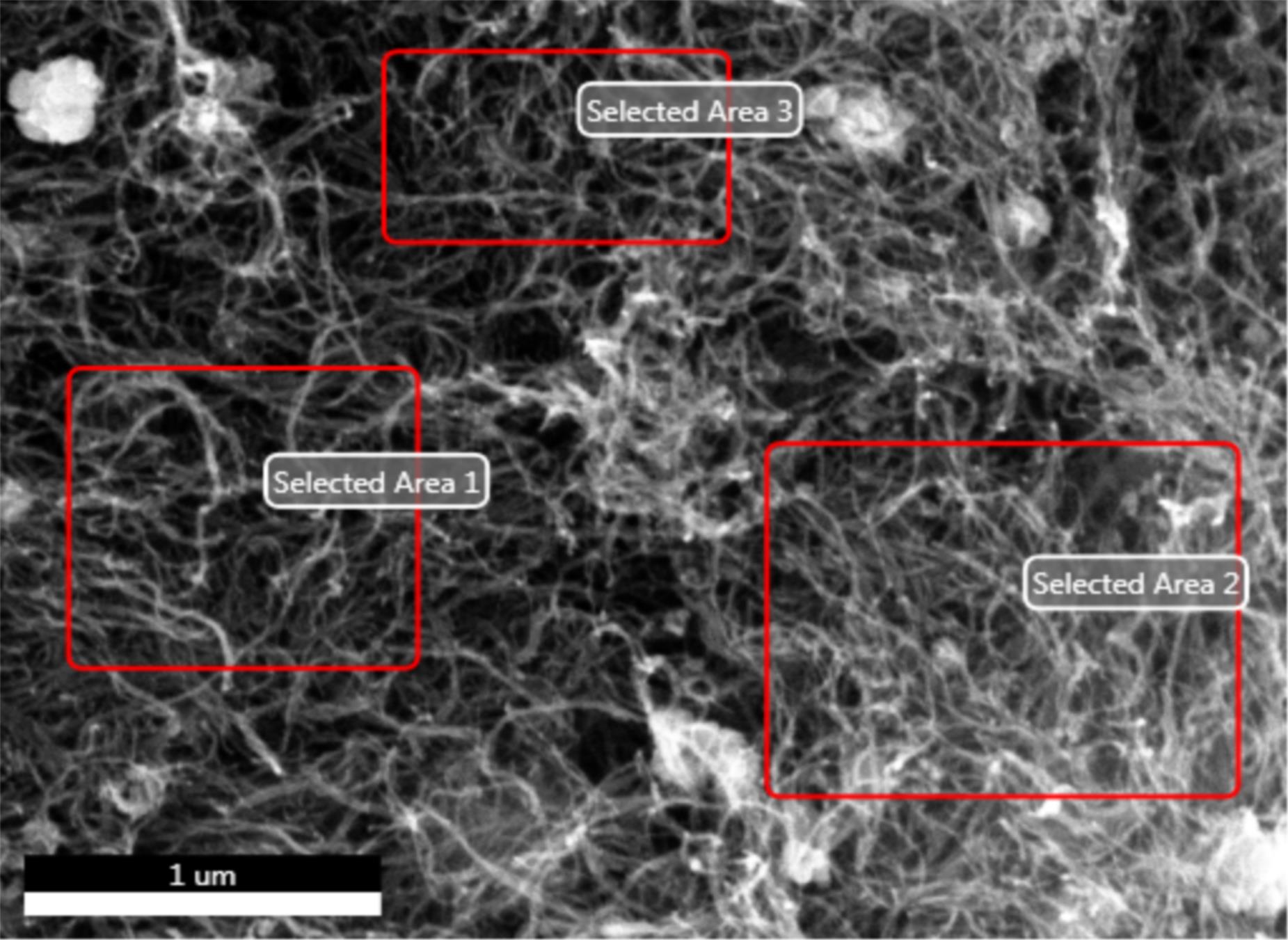 | | | | | | | | | | | | | | | | | | | | | | | | | | | |  |  |  |  |  |  |  |  |  |  |  |  |  |  |  |
|  |  |  |  |  |  |  |  |  |  |  |  |  |  |  |  |  |  |  |  |  |  |  |  |  |  |  |  |  |  |  |  |  |  |  |  |  |  |  |  |  |  |  |
| **EDAX TEAM** | | | | | | | | | | | | | | | | | | | | | | | | | | | | | | | | | | | | |  |  |  |  |  |  |
|  |  |  |  |  |  |  |  |  |  |  |  |  |  |  |  |  |  |  |  |  |  |  |  |  |  |  |  |  |  |  |  |  |  |  |  |  |  |  |  |  |  |  |
|  |  |  | | | |  |  | |  | |  |  | | |  | | | |  |  |  |  |  |  |  |  |  | | |  |  |  |  |  | |  |  |  |  |  |  |  |
|  |  |  | | | |  |  | |  | |  |  | | |  | | | |  |  |  |  |  |  |  |  |  | | |  |  |  |  |  | |  |  |  |  |  |  |  |
| 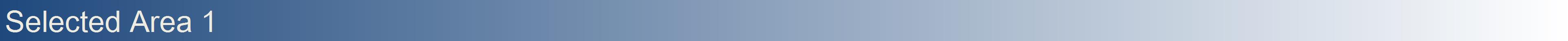 | | | | | | | | | | | | | | | | | | | | | | | | | | | | | | | | | | | | | | | | | | |
|  |  |  | | | |  |  | |  | |  |  | | |  | | | |  |  |  |  |  |  |  |  |  | | |  |  |  |  |  | |  |  |  |  |  |  |  |
|  |  |  | | | |  |  | |  | |  |  | | |  | | | |  |  |  |  |  |  |  |  |  | | |  |  |  |  |  | |  |  |  |  |  |  |  |
|  |  |  | | | |  | Mag: | | | | 100000 | | | |  | | | | Takeoff: |  | 74.7 | |  |  |  | Live Time(s): | | | | |  | 29.7 |  |  | |  |  |  |  |  |  |  |
|  |  |  | | | |  |  | |  | |  |  | | |  | | | |  |  |  |  |  |  |  |  |  | | |  |  |  |  |  | |  |  |  |  |  |  |  |
|  | **Selected Area 1 - EDS** | | | | | | | | | | | | | | | | | | | | | | | | | | | | | | | | | | | | | | | |  |  |
|  | 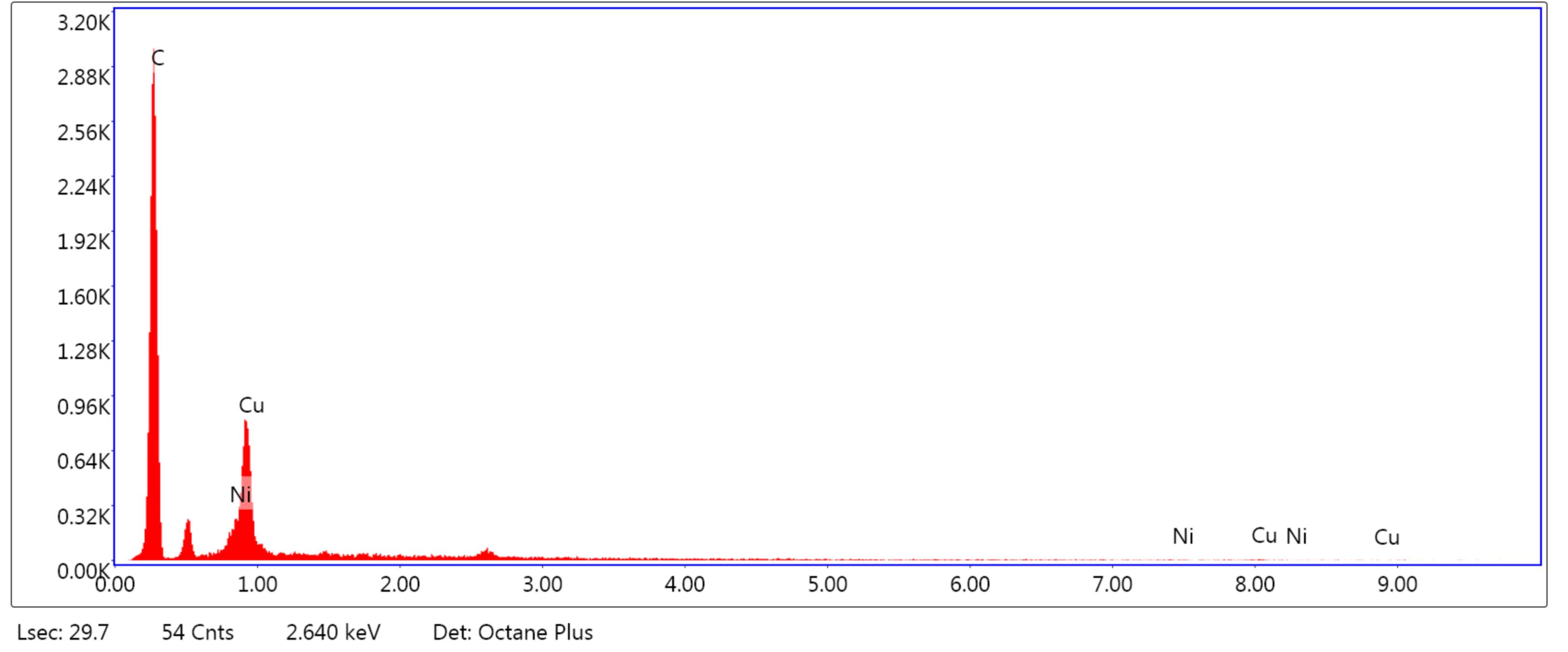 | | | | | | | | | | | | | | | | | | | | | | | | | | | | | | | | | | | | | | | |  |  |
|  |  |  |  |  |  |  |  |  |  |  |  |  |  |  |  |  |  |  |  |  |  |  |  |  |  |  |  |  |  |  |  |  |  |  |  |  |  |  |  |  |  |  |
|  |  |  |  |  |  |  |  |  |  |  |  |  |  |  |  |  |  |  |  |  |  |  |  |  |  |  |  |  |  |  |  |  |  |  |  |  |  |  |  |  |  |  |
|  |  |  | | | |  |  | |  | |  |  | | |  | | | |  |  |  |  |  |  |  |  |  | | |  |  |  |  |  | |  |  |  |  |  |  |  |
| **eZAF Smart Quant Results** | | | | | | | | | | | | | | | | | | | | | | | | | | | | | | | | | | | | | | | | | |  |
|  |  |  | | | |  |  | |  | |  |  | | |  | | | |  |  |  |  |  |  |  |  |  | | |  |  |  |  |  | |  |  |  |  |  |  |  |
|  |  |  | | | |  |  | |  | |  |  | | |  | | | |  |  |  | Element | | | Weight % | | | | | Atomic % | | | |  | |  |  |  |  |  |  |  |
|  |  |  | | | |  |  | |  | |  |  | | |  | | | |  |  |  | C K | | | 72.50 | | | | | 93.24 | | | |  | |  |  |  |  |  |  |  |
|  |  |  | | | |  |  | |  | |  |  | | |  | | | |  |  |  | NiL | | | 3.52 | | | | | 0.93 | | | |  | |  |  |  |  |  |  |  |
|  |  |  | | | |  |  | |  | |  |  | | |  | | | |  |  |  | CuL | | | 23.98 | | | | | 5.83 | | | |  | |  |  |  |  |  |  |  |
|  |  |  | | | |  |  | |  | |  |  | | |  | | | |  |  |  |  |  |  |  |  |  | | |  |  |  |  |  | |  |  |  |  |  |  |  |

**Figure S3.** SEM-EDX figure of NiCu @ MWCNT nanohybrids

**
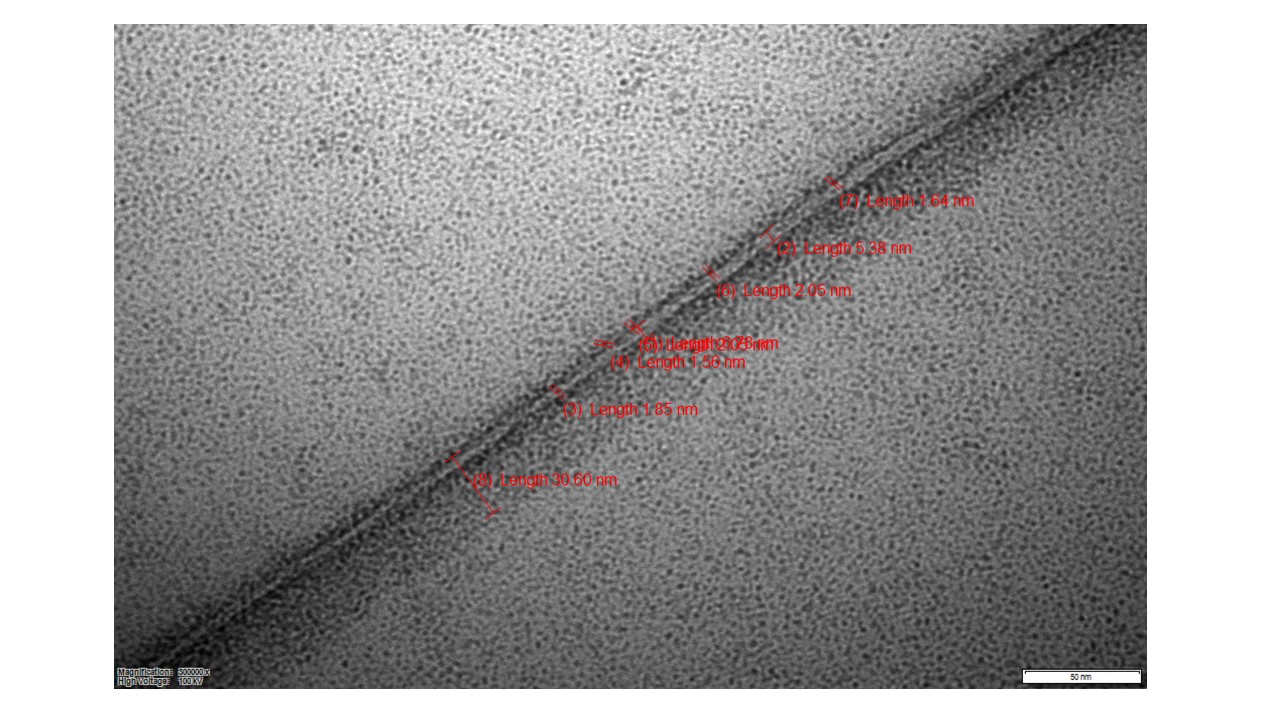
**

**Figure S4.** TEM image of NiCu@MWCNT nanohybrids

**Preparation and application of the Knoevenagel condensation studies of aryl aldehydes**

4 ml solution of water/methanol (1:1) containing 4 mg NiCu@MWCNT nanohybrids was taken place in an ultrasonic bath for 30 s, and at room temperature 1.0 mmol of malononitrile was transferred into the resulting solution (during stirring), and then the slurry was closed. The reaction occurred in the solution was controlled by the thin layer chromatography (TLC) formed during stirring. Almost the whole reaction was completed in 10-35 minutes, and then the slurry was centrifugated to separate the catalyst at 7500 rpm. The obtained nanohybrid was washed several times using water and methanol, dried in vacuum at room temperature. the purification of the solid sample was done using a chromatography system containing a ratio of 1:9 EtOAc/hexane. ^1^H and ^13^C NMR spectra were taken with CDCl_3_ solvent and yields were determined using spectra.

**Table S1** Comparison of designed catalytic system with recent published works about the Knoevenagel condensation of benzaldehyde with malononitrile

| **Catalyst** | **Conditions** | **Temp.**  ^o^**C** | **Time, min** | **Yield^a^, %** |
| --- | --- | --- | --- | --- |
| SiO_2_-Pr-SO_3_H^1^ | Benzaldehyde (1 mmol), malononitrile (1 mmol), catalyst (0.1 g) | 120 | 240 | 95 |
| Cd(II)-based coordination polymer^2^ | Benzaldehyde (0.5 mmol), malononitrile (1 mmol), catalyst (3 mol%), THF (1 ml) | 50 | 240 | 94 |
| Ni(II)-based coordination polymer^3^ | Benzaldehyde (10.4 mmol), malononitrile (10.4 mmol), catalyst (5% by weight with respect to the amount of benzaldehyde), DCM | 60 | 120 | 100 |
| Zn(II)-based coordination polymer^4^ | Benzaldehyde (0.2 mmol), malononitrile (0.6 mmol), catalyst (0.004 mmol) | 25 | 180 | 99 |
| MOF-NH_2_^5^ | Benzaldehyde (1 mmol), malononitrile (1 mmol), catalyst (0.13 mmol), DMF (6 ml) | 80 | 270 | 51 |
| PMOV1^6^ | Benzaldehyde (10 mmol), malononitrile (10 mmol), catalyst (2 mol%) | 70 | 45 | 86 |
| **NiCu NPs@MWCNT (this study)** | Benzaldehyde (1.0 mmol), malononitrile (1.0 mmol), catalyst (0.033 mmol%), water/methanol (v/v=1/1) | 25 | 15 | 95 |

^a^isolated yield

**References**

1. G. Mohammadi Ziarani, A. Badiel, Z. Dashtianeh, P. Hajiabbasi, Rev. Roum. Chim. 58 (2013) 765-772.
2. A. Karmakar, A. Paul, K. T. Mahmudov, M. F. C. G. da Silva, A. J. L. Pombeiro, New. J. Chem. 40 (2016) 1535-1546.
3. R. A. Agarwal, S. Mukherjee, Polyhedron 105 (2016) 228-237.
4. L. Ma, X. Wang, D. Deng, F. Luo, B. Ji, J. Zhang, J. Mater. Chem. A. 3 (2015) 20210-20217.
5. A. Taher, D.-J. Lee, B.-K. Lee, I.-M. Lee, Synlett 27 (2016) 1433-1437.
6. B. Viswanadham, P. Jhansi, K. V. R. Chary, H. B. Friedrich, S. Singh, Catal. Lett. 146 (2016) 364-372.
7. ***2-(3,4,5-trimethoxybenzylidene)malononitrile (2):*** ^1^H NMR (400 MHz, CDCl_3_): *δ* 7.65 (s, 1 H), 7.19 (s, 2H), 3.98 (s, 3H), 3.91 (s, 6H). ^13^C NMR (100 MHz, CDCl_3_): *δ* 159.3, 153.4, 144.1, 125.9, 113.9, 113.2, 108.3, 80.6, 61.2, 56.4.
8. ***2-(4-hydroxybenzylidene)malononitrile (4):*** ^1^H NMR (400 MHz, CDCl_3_): *δ* 7.88 (d, *J* = 8.6 Hz, 2H), 7.64 (s, 1H), 6.96 (d, *J* = 8.8 Hz, 2H). ^13^C NMR (100 MHz, CDCl_3_): *δ* 158.6, 133.3, 116.7, 112.7, 111.4, 88.7.
9. ***2-(4-methylbenzylidene)malononitrile (6*):** ^1^H NMR (400 MHz, CDCl_3_): *δ* 7.80 (d, *J* = 8.2 Hz, 2H), 7.71 (s, 1H), 7.32 (d, *J* = 8.2 Hz, 2H), 2.44 (s, 3H). ^13^C NMR (100 MHz, CDCl_3_): *δ* 159.9, 146.5, 131.0, 114.1, 112.9, 108.8, 81.3, 22.2.
10. ***2-(2-methylbenzylidene)malononitrile (8):*** ^1^H NMR (400 MHz, CDCl_3_): *δ* 8.09 (s, 1H), 8.07 (d, *J* = 7.9 Hz, 1H), 7.48 (m, 1H), 7.33 (dd, *J* = 16.3, 7.9 Hz, 2H), 2.46 (s, 3H). ^13^C NMR (100 MHz, CDCl_3_): *δ* 158.4, 139.8, 134.4, 131.7, 129.9, 128.3, 112.5, 108.8, 84.0, 19.9.
11. ***2-(4-nitrobenzylidene)malononitrile (10):*** ^1^H NMR (400 MHz, CDCl_3_): *δ* 8.39 (d, *J* = 8.8 Hz, 2H), 8.07 (d, *J* = 8.8 Hz, 2H), 7.87 (s, 1H). ^13^C NMR (100 MHz, CDCl_3_): *δ* 156.7, 135.7, 131.3, 124.6, 112.8, 111.6, 87.6.
12. ***2-(4-(trifluoromethyl)benzylidene)malononitrile (12):*** ^1^H NMR (400 MHz, CDCl_3_): *δ* 8.00 (d, *J* = 8.2 Hz, 2H), 7.84 (s, 1H), 7.80 (d, *J* = 8.2 Hz, 2H). ^13^C NMR (100 MHz, CDCl_3_): *δ* 158.2,133.8, 130.9, 126.7, 121.8, 113.0, 111.9, 86.1.
13. ***2-(4-fluorobenzylidene)malononitrile (14):*** ^1^H NMR (400 MHz, CDCl_3_): *δ* 7.96 (m, 2H), 7.73 (s, 1H), 7.22 (m, 2H). ^13^C NMR (100 MHz, CDCl_3_): *δ* 158.4, 133.5, 127.4, 117.6, 113.6, 112.6, 82.5.
14. ***2-(2-fluorobenzylidene)malononitrile (16):*** ^1^H NMR (400 MHz, CDCl_3_): *δ* 8.27 (m, 1H), 8.09 (s, 1H), 7.63 (m, 1H), 7.31 (m, 1H), 7.22 (m, 1H). ^13^C NMR (100 MHz, CDCl_3_): *δ* 162.7, 160.1, 136.9, 128.7, 119.4, 116.6, 113.5, 108.7, 84,6.
15. ***2-(4-chlorobenzylidene)malononitrile (18):*** ^1^H NMR (400 MHz, CDCl_3_): *δ* 7.85 (d, *J* = 8.6 Hz, 2H), 7.73 (s, 1H), 7.52 (d, *J* = 8.6 Hz, 2H). ^13^C NMR (100 MHz, CDCl_3_): *δ* 158.2, 141.1, 131.8, 130.1, 129.2, 113.4, 112.3, 83.4.
16. ***2-(4-iodobenzylidene)malononitrile (20):*** ^1^H NMR (400 MHz, CDCl_3_): *δ* 7.91 (d, *J* = 8.4 Hz, 2H), 7.69 (s, 1H), 7.60 (d, *J* = 8.5 Hz, 2H). ^13^C NMR (100 MHz, CDCl_3_): *δ* 158.6, 139.0, 131.5, 130.1, 113.4, 112.3, 83.6.
17. ***2-(anthracen-9-ylmethylene)malononitrile (22):*** ^1^H NMR (400 MHz, CDCl_3_): *δ* 8.95 (s, 1H), 8.65 (s, 1H), 8.10 (d, *J* = 8.4 Hz, 2H), 7.93 (d, *J* = 8.8 Hz, 2H), 7.68 (m, 2H), 7.59 (m, 2H). ^13^C NMR (100 MHz, CDCl_3_): *δ* 160.5, 132.5, 130.9, 129.5, 129.1, 128.3, 126.0, 123.8, 123.4, 112.9, 111.3, 92.4.
18. ***2-benzylidenemalononitrile (24):*** ^1^H NMR (400 MHz, CDCl_3_): *δ* 7.91 (d, *J* = 7.8 Hz, 1H), 7.78 (s, 1H), 7.63 (m, 1H), 7.54 (m, 1H). ^13^C NMR (100 MHz, CDCl_3_): *δ* 159.9, 134.6, 130.9, 130.7, 129.6, 113.7, 112.5, 82.9.
19. ***2-(2-Furanylmethylene)malononitrile (26):*** ^1^H NMR (400 MHz, *d_6_*-DMSO): *δ* 8.35-8.27 (m, 2H), 7.46 (d, *J* = 3.7 Hz, 1H), 6.95-6.90 (m, 1H). ^13^C NMR (100 MHz, *d_6_*-DMSO): *δ* 151.7, 148.5, 144.9, 126.3, 115.2, 113.8, 75.4.
20. ***2-Butylidenemalononitrile (28):*** ^1^H NMR (400 MHz, CDCl_3_): *δ* 7.37 (t, *J* = 8.0 Hz, 1H), 2.63-2.52 (m, 2H), 1.69-1.56 (m, 2H), 1.02 (t, *J* = 7.4 Hz, 3H). ^13^C NMR (100 MHz, CDCl_3_): *δ* 169.90, 112.19, 110.65, 89.89, 34.68, 21.05, 13.56.

**^1^H/^13^C-NMR Spectra for BMN Derivatives**

**2-(3,4,5-trimethoxybenzylidene)malononitrile (2):** ^1^H NMR (400 MHz, CDCl_3_): *δ* 7.65 (s, 1 H), 7.19 (s, 2H), 3.98 (s, 3H), 3.91 (s, 6H). ^13^C NMR (100 MHz, CDCl_3_): *δ* 159.3, 153.4, 144.1, 125.9, 113.9, 113.2, 108.3, 80.6, 61.2, 56.4.

**2-(4-hydroxybenzylidene)malononitrile (4):** ^1^H NMR (400 MHz, CDCl_3_): *δ* 7.88 (d, *J* = 8.6 Hz, 2H), 7.64 (s, 1H), 6.96 (d, *J* = 8.8 Hz, 2H). ^13^C NMR (100 MHz, CDCl_3_): *δ* 158.6, 133.3, 116.7, 112.7, 111.4, 88.7.

**
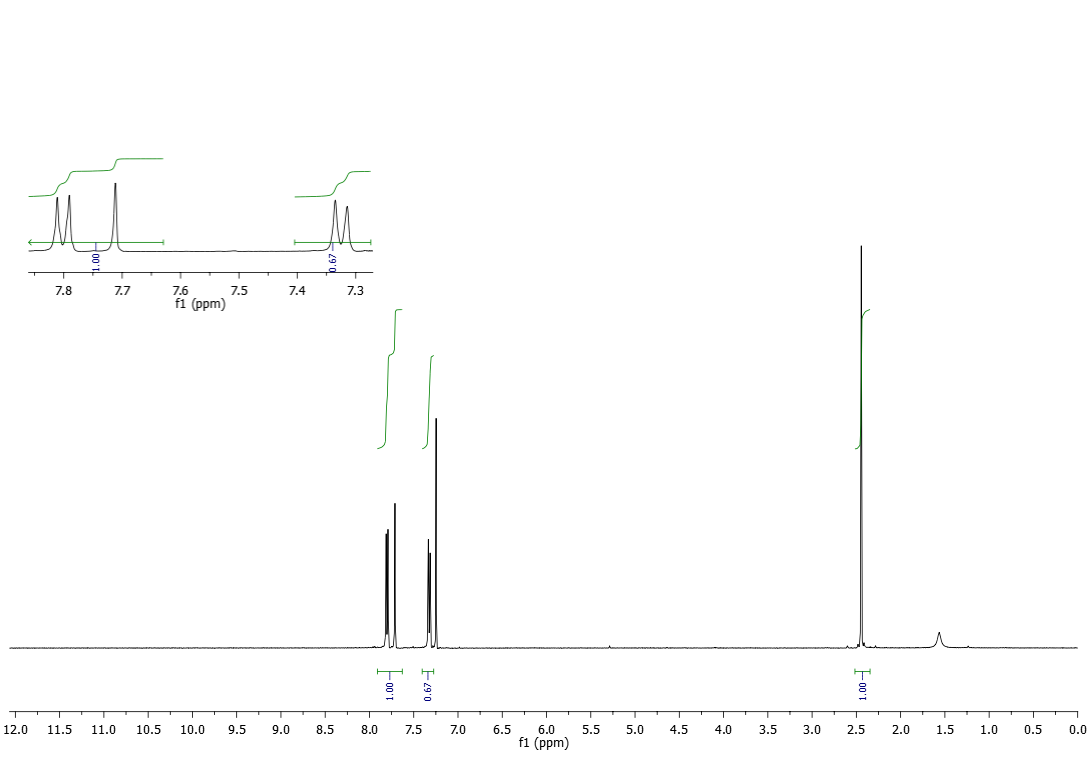
**

**2-(4-methylbenzylidene)malononitrile (6):** ^1^H NMR (400 MHz, CDCl_3_): *δ* 7.80 (d, *J* = 8.2 Hz, 2H), 7.71 (s, 1H), 7.32 (d, *J* = 8.2 Hz, 2H), 2.44 (s, 3H). ^13^C NMR (100 MHz, CDCl_3_): *δ* 159.9, 146.5, 131.0, 114.1, 112.9, 108.8, 81.3, 22.2.


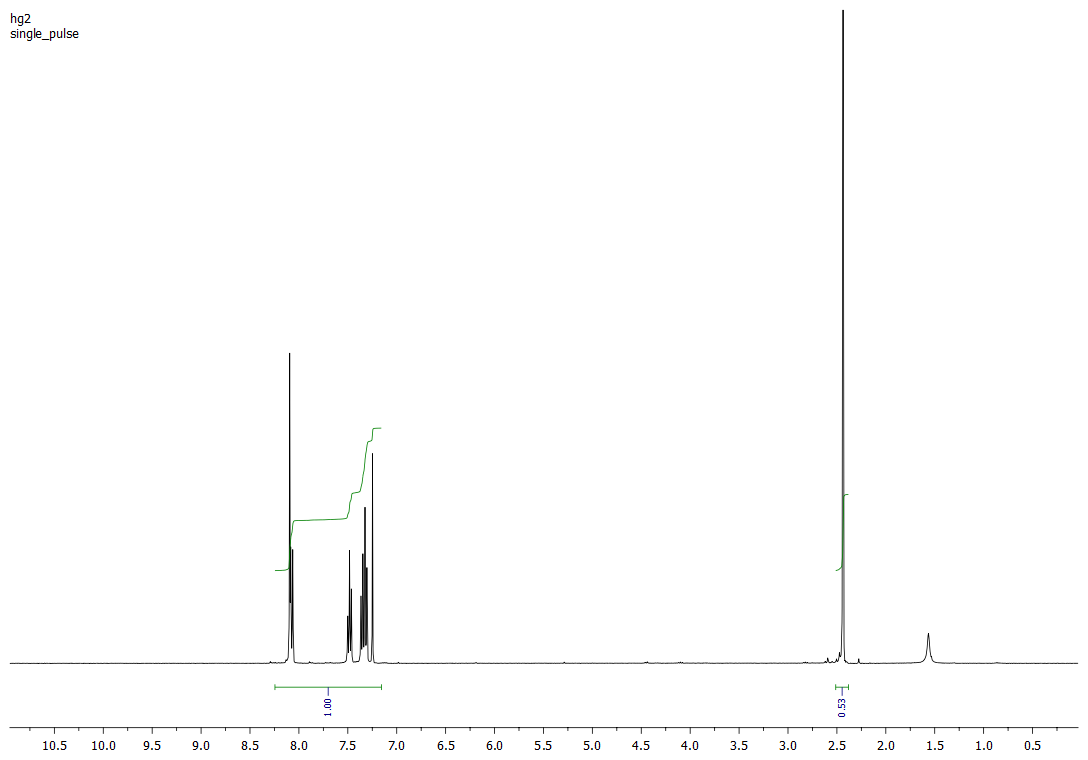

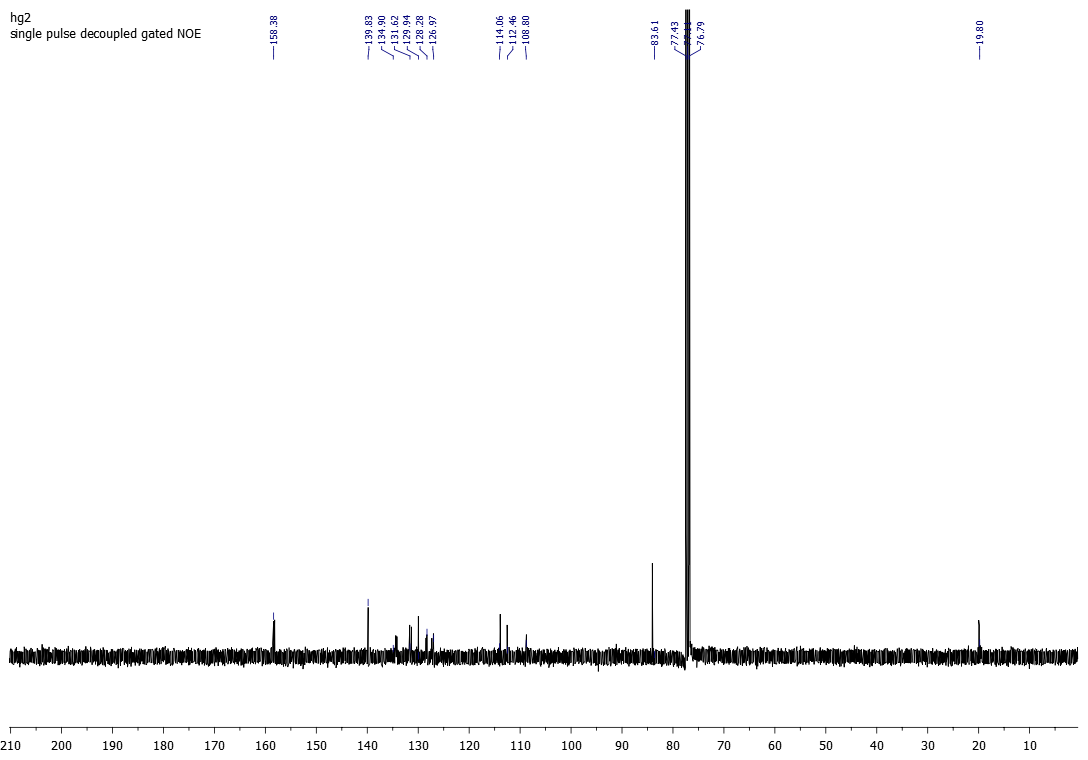


**2-(2-methylbenzylidene)malononitrile (8):** ^1^H NMR (400 MHz, CDCl_3_): *δ* 8.09 (s, 1H), 8.07 (d, *J* = 7.9 Hz, 1H), 7.48 (m, 1H), 7.33 (dd, *J* = 16.3, 7.9 Hz, 2H), 2.46 (s, 3H). ^13^C NMR (100 MHz, CDCl_3_): *δ* 158.4, 139.8, 134.4, 131.7, 129.9, 128.3, 112.5, 108.8, 84.0, 19.9.

**
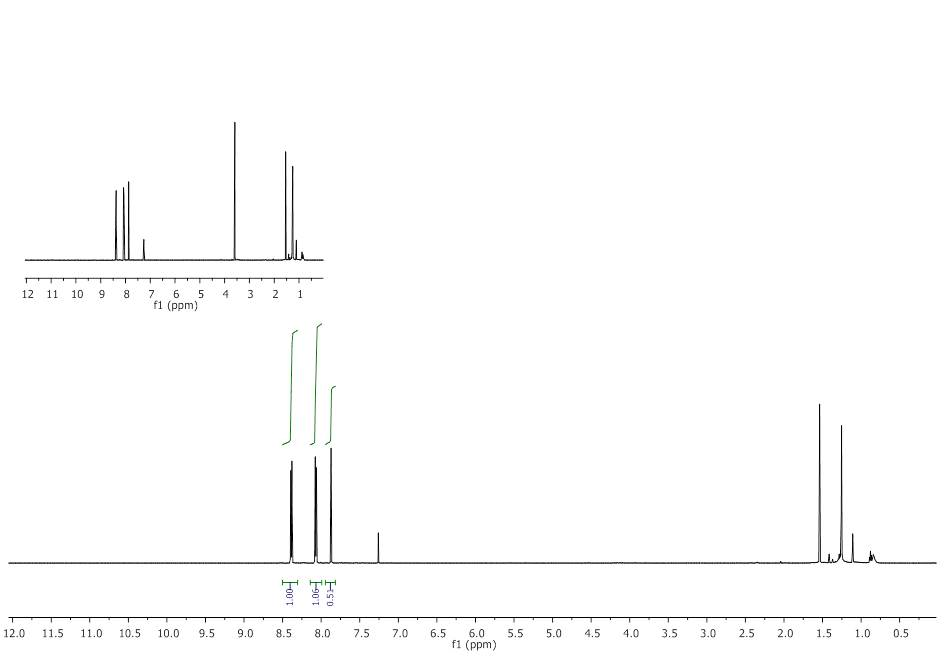
**

**2-(4-nitrobenzylidene)malononitrile (10):** ^1^H NMR (400 MHz, CDCl_3_): *δ* 8.39 (d, *J* = 8.8 Hz, 2H), 8.07 (d, *J* = 8.8 Hz, 2H), 7.87 (s, 1H). ^13^C NMR (100 MHz, CDCl_3_): *δ* 156.7, 135.7, 131.3, 124.6, 112.8, 111.6, 87.6.

**2-(4-(trifluoromethyl)benzylidene)malononitrile (12):** ^1^H NMR (400 MHz, CDCl_3_): *δ* 8.00 (d, *J* = 8.2 Hz, 2H), 7.84 (s, 1H), 7.80 (d, *J* = 8.2 Hz, 2H). ^13^C NMR (100 MHz, CDCl_3_): *δ* 158.2,133.8, 130.9, 126.7, 121.8, 113.0, 111.9, 86.1.

**2-(4-fluorobenzylidene)malononitrile (14):** ^1^H NMR (400 MHz, CDCl_3_): *δ* 7.96 (m, 2H), 7.73 (s, 1H), 7.22 (m, 2H). ^13^C NMR (100 MHz, CDCl_3_): *δ* 158.4, 133.5, 127.4, 117.6, 113.6, 112.6, 82.5.

**2-(2-fluorobenzylidene)malononitrile (16):** ^1^H NMR (400 MHz, CDCl_3_): *δ* 8.27 (m, 1H), 8.09 (s, 1H), 7.63 (m, 1H), 7.31 (m, 1H), 7.22 (m, 1H). ^13^C NMR (100 MHz, CDCl_3_): *δ* 162.7, 160.1, 136.9, 128.7, 119.4, 116.6, 113.5, 108.7, 84,6.


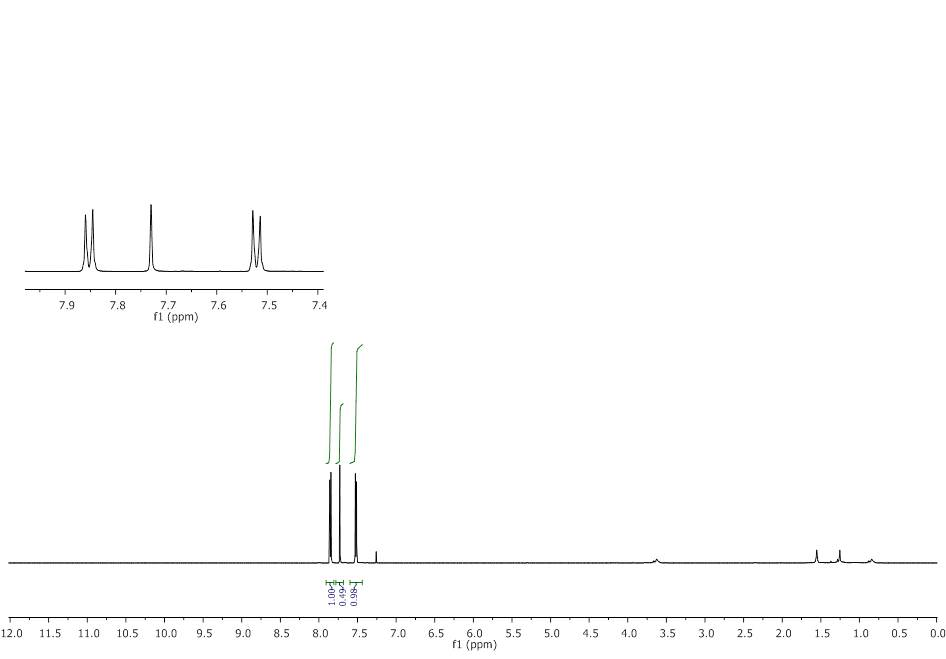

**2-(4-chlorobenzylidene)malononitrile (18):** ^1^H NMR (400 MHz, CDCl_3_): *δ* 7.85 (d, *J* = 8.6 Hz, 2H), 7.73 (s, 1H), 7.52 (d, *J* = 8.6 Hz, 2H). ^13^C NMR (100 MHz, CDCl_3_): *δ* 158.2, 141.1, 131.8, 130.1, 129.2, 113.4, 112.3, 83.4.

**
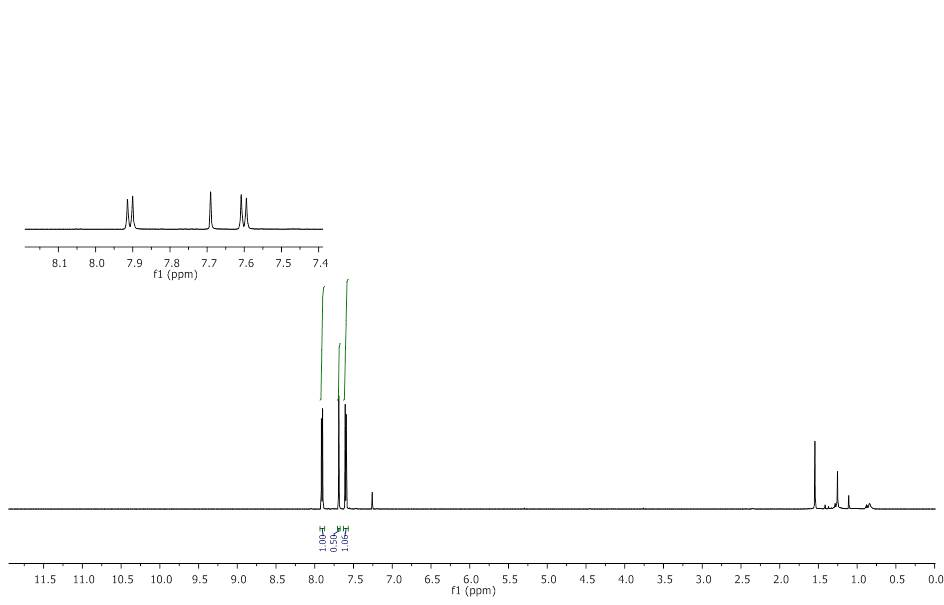
**

**2-(4-iodobenzylidene)malononitrile (20):** ^1^H NMR (400 MHz, CDCl_3_): *δ* 7.91 (d, *J* = 8.4 Hz, 2H), 7.69 (s, 1H), 7.60 (d, *J* = 8.5 Hz, 2H). ^13^C NMR (100 MHz, CDCl_3_): *δ* 158.6, 139.0, 131.5, 130.1, 113.4, 112.3, 83.6.

**2-(anthracen-9-ylmethylene)malononitrile (22):** ^1^H NMR (400 MHz, CDCl_3_): *δ* 8.95 (s, 1H), 8.65 (s, 1H), 8.10 (d, *J* = 8.4 Hz, 2H), 7.93 (d, *J* = 8.8 Hz, 2H), 7.68 (m, 2H), 7.59 (m, 2H). ^13^C NMR (100 MHz, CDCl_3_): *δ* 160.5, 132.5, 130.9, 129.5, 129.1, 128.3, 126.0, 123.8, 123.4, 112.9, 111.3, 92.4.

**2-benzylidenemalononitrile (24):** ^1^H NMR (400 MHz, CDCl_3_): *δ* 7.91 (d, *J* = 7.8 Hz, 1H), 7.78 (s, 1H), 7.63 (m, 1H), 7.54 (m, 1H). ^13^C NMR (100 MHz, CDCl_3_): *δ* 159.9, 134.6, 130.9, 130.7, 129.6, 113.7, 112.5, 82.9.

**
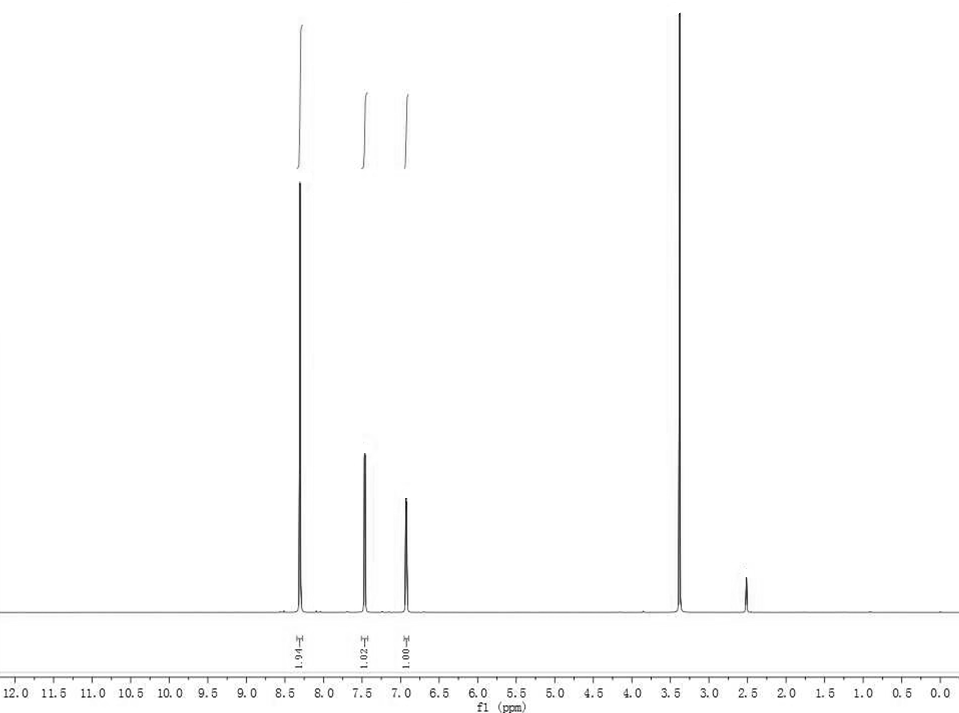
**


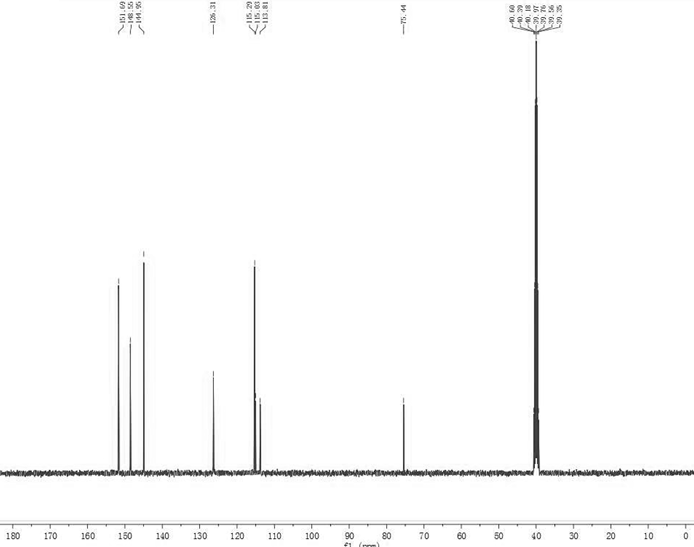


**2-(2-Furanylmethylene)malononitrile (26):** ^1^H NMR (400 MHz, *d_6_*-DMSO): *δ* 8.35-8.27 (m, 2H), 7.46 (d, *J* = 3.7 Hz, 1H), 6.95-6.90 (m, 1H). ^13^C NMR (100 MHz, *d_6_*-DMSO): *δ* 151.7, 148.5, 144.9, 126.3, 115.2, 113.8, 75.4.


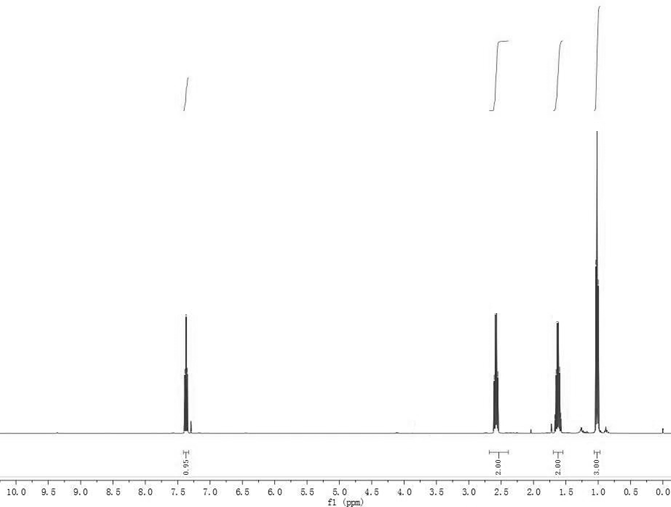


**
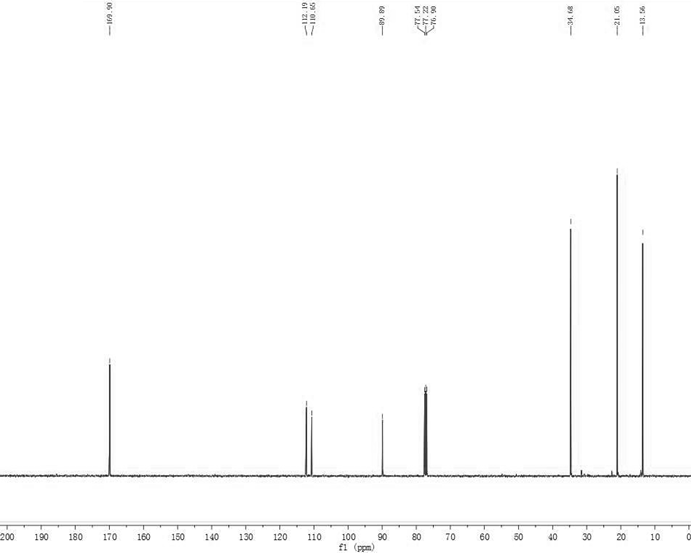
2-Butylidenemalononitrile (28):** ^1^H NMR (400 MHz, CDCl_3_): *δ* 7.37 (t, *J* = 8.0 Hz, 1H), 2.63-2.52 (m, 2H), 1.69-1.56 (m, 2H), 1.02 (t, *J* = 7.4 Hz, 3H). ^13^C NMR (100 MHz, CDCl_3_): *δ* 169.90, 112.19, 110.65, 89.89, 34.68, 21.05, 13.56.
